# Supplementary material for: Identification of photoperiod-induced specific miRNAs in the adrenal glands of Sunite sheep (Ovis aries)
Source: Front Vet Sci. 2022 Jul 22;9:888207. doi: 10.3389/fvets.2022.888207 (PMC9354845; doi:10.3389/fvets.2022.888207)
Supplement: Supplementary file 1 [file Data_Sheet_1.ZIP › Supplementary file/miRDeep2 file.zip!/miRDeep2 file/Know_Structure/LP42A2/oar-mir-30d.pdf]

miRBase precursor : oar-mir-30d
Total read count : 196905
oar-miR-30d read count : 196058
remaining reads : 847

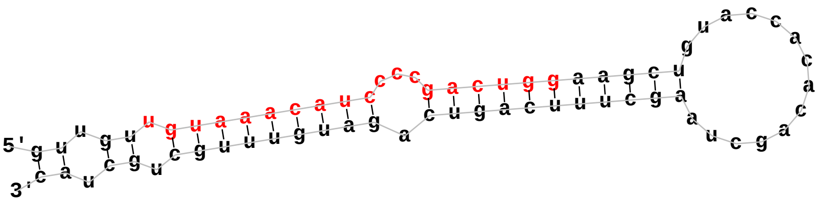

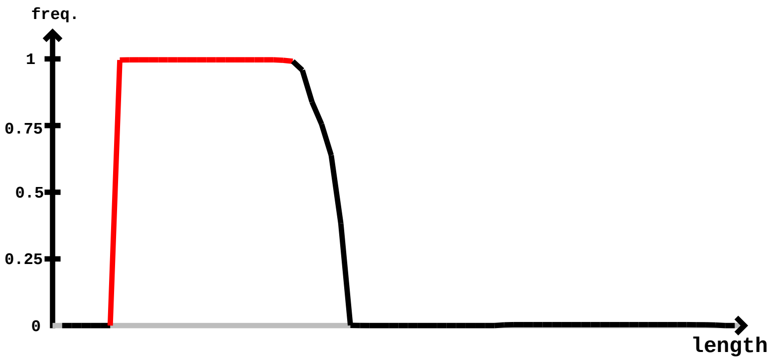

oar-miR-30d

```
5'- guuguuguaaacauccccgacuggaagcuguaccacacagcuaagcuuucagucagauguuugcugcuac   -3'   exp
    ...((.(((((((((...((((((((((................)))))))))).))))))))).))...   reads   mm   sample
    ....uuguaaacauccccgacugga.............................................   2       0    seq
    ....uuguaaacauccccgacuggaagc..........................................   1       0    seq
    ....uuguaaacauccccgacuggaagcu.........................................   2       0    seq
    ....uuguaaacauccccgacuggaagcugu.......................................   1       0    seq
    .....uguaaacauccccgacu................................................   324     0    seq
    .....uguaaacaucccUgacu................................................   10      1    seq
    .....uguaaacauccccgGcu................................................   3       1    seq
    .....uguaaacaucUccgacu................................................   1       1    seq
    .....uguaaacauccccgacug...............................................   728     0    seq
    .....uguaaacaAccccgacug...............................................   2       1    seq
    .....uguaaacauccccgacugg..............................................   6625    0    seq
    .....uguaaacauccccgacugga.............................................   23383   0    seq
    .....uguaaacauccccgacuggaa............................................   16407   0    seq
    .....uguaaacauccccgacuggaag...........................................   22970   0    seq
    .....uguaaacauccccgacuggaagc..........................................   50200   0    seq
    .....uguaaacauccccgacuggaagcu.........................................   75269   0    seq
    .....uguaaacauccccgacuggaagcug........................................   102     0    seq
    .....uguaaacauccccgacuggaagcugu.......................................   42      0    seq
    .....uguaaacauccccgacuggaagcuguacc....................................   2       0    seq
    .....uguaaacauccccgacuggaagcuguacca...................................   1       0    seq
    ......guaaacauccccgacugg..............................................   3       0    seq
    ......guaaacauccccgacugga.............................................   6       0    seq
    ......guaaacauccccgacuggaa............................................   7       0    seq
    ......guaaacauccccgacuggaag...........................................   13      0    seq
    ......guaaacauccccgacuggaagc..........................................   31      0    seq
    ......guaaacauccccgacuggaagcu.........................................   66      0    seq
    .......uaaacauccccgacugga.............................................   1       0    seq
    .......uaaacauccccgacuggaa............................................   1       0    seq
    .......uaaacauccccgacuggaagcu.........................................   2       0    seq
    .......uaaacauccccgacuggaagcug........................................   1       0    seq
    ........aaacauccccgacuggaagc..........................................   1       0    seq
    .............................................cuuucagucagauguuugc......   16      0    seq
    .............................................cuuucagucagauguuugcu.....   55      0    seq
    .............................................cuuucagucagauguuugcug....   48      0    seq
    .............................................cuuucagucagauguuugcugc...   144     0    seq
```

oar-miR-30d

```
guuguuguaaacauccccgacuggaagcuguaccacacagcuaagcuuucagucagauguuugcugcuac
.............................................cuuucagucagauguuugcugcu..    254    0    seq
.............................................cuuucagucagauguuugcugcua.    3      0    seq
..............................................uuucagucagauguuugc......    1      0    seq
..............................................uuucagucagauguuugcu.....    6      0    seq
..............................................uuucagucagauguuugcug....    8      0    seq
..............................................uuucagucagauguuugcugc...    30     0    seq
..............................................uuucagucagauguuugcugcu..    127    0    seq
..............................................uuucagucagauguuugcugcua.    2      0    seq
..............................................uuucagucagauguuugcugcuac    1      0    seq
...............................................uucagucagauguuugcugc...    3      0    seq
```
